# Aerobic Exercise and PI3K Inhibitor Ameliorate Obesity Cardiomyopathy by Alleviating Pyroptosis in Middle-Aged Mice

**DOI:** 10.3390/ijms26104935

**Published:** 2025-05-21

**Authors:** Bojun Yang, Jiahao Xu, Xiaoyan Dao, Yu Huang, Jiling Liang, Jielun Huang, Bo Gou, Hanyu Yan, Ning Chen, Jingjing Fan

**Affiliations:** Hubei Key Laboratory of Exercise Training and Monitoring, College of Sports Medicine, Wuhan Sports University, Wuhan 430079, China; yangbojun2021@163.com (B.Y.); nchen510@gmail.com (N.C.)

**Keywords:** obesity cardiomyopathy, PI3K/AKT, NLRP3 inflammasome, pyroptosis

## Abstract

Obesity cardiomyopathy (OCM) represents a rapidly growing health concern globally, characterized by metabolic, structural, and functional abnormalities of the heart. Current research has demonstrated that inflammation plays a pivotal role in obesity-induced cardiomyopathy, and that regular exercise can ameliorate lipid disturbances and inflammatory abnormalities effectively. However, the underlying mechanisms are not fully elucidated. We investigated the effects of an 8-week aerobic exercise intervention on myocardial structure, function, and inflammation in HFD-induced obese mice. The results revealed that aerobic exercise alleviated myocardium pyroptosis and inflammation by down-regulating the PI3K/AKT signaling pathway. Furthermore, the inhibition of the PI3K pathway by LY294002, coupled with exercise, attenuated and suppressed HFD-induced myocardial impairments, inflammation, and pyroptosis, with a synergistic effect. Based on these findings, we concluded that eight weeks of aerobic exercise synergizes with the inhibition of PI3K through inflammatory and pyroptosis mechanisms to improve obesity-associated myocardial remodeling and dysfunction. Therefore, long-term regular aerobic exercise represents a potential strategy in the treatment of OCM.

## 1. Introduction

The global incidence of obesity continues to rise, with an increasing proportion of adults becoming obese as living standards improve and energy intake increases. This population is at an elevated risk of developing chronic diseases. Obesity is widely recognized as a metabolic disorder strongly associated with health problems such as hyperglycemia, hypertension, and cardiovascular disease [1]. OCM is a heart condition that is characterized by metabolic, structural, and functional abnormalities in the heart, which is independent of diabetes, hypertension, coronary artery disease, and other cardiovascular conditions [2]. The underlying pathogenesis of OCM is not fully understood. Thus, elucidation of the pathogenesis and development of effective interventions for the treatment of this disease are urgent priorities.

Programmed cell death encompasses a variety of forms, primarily including apoptosis, pyroptosis, necroptosis, ferroptosis, and cuproptosis. In OCM, one or multiple forms of cell death may participate in the initiation and progression of the disease [3]. Recently, pyroptosis, characterized by an inflammatory response, has been found to play an important role in obesity-induced associated cardiomyopathy. Studies have found that chronic inflammation plays a crucial role in obesity-related cardiomyopathy, activating the NOD-like receptor protein 3 (NLRP3) inflammasome and thereby triggering pyroptosis [4,5]. Under inflammatory conditions, the NLRP3 inflammasome, composed of NLRP3, apoptosis-associated speck-like protein (ASC), and the effector molecule pro-cysteinyl aspartate specific proteinase-1 (Pro-caspase-1), serves as a key signaling platform that integrates both endogenous and exogenous stimulatory signals by activating the Caspase-1-mediated proteolytic process, which promotes the formation of mature Interleukin-1beta (IL-1β) and Interleukin-18 (IL-18). Meanwhile, activated Caspase-1 cleaves Gasdermin-D (GSDMD) to generate the N-terminal fragment (GSDMD-N), which triggers the formation of transmembrane pores. The release of active IL-1β and IL-18, together with GSDMD-N, then regulates the recruitment and activation of immune cells to eliminate harmful stimuli [6,7,8]. Despite recent studies highlighting the pivotal role of NLRP3-induced pyroptosis in obesity-induced cardiac dysfunction, the underlying molecular mechanisms remain poorly understood. The Phosphatidylinositol 3-kinase/protein kinase (PI3K/AKT) signaling pathway plays a crucial role in regulating various cellular metabolic processes, such as cell proliferation, survival, and protein synthesis [9]. In cardiovascular diseases, this pathway is pivotal in modulating cardiomyocyte growth and proliferation, angiogenesis, and inflammatory responses. Recent studies have demonstrated a close association between the PI3K/AKT pathway and inflammatory responses, highlighting its role in regulating the NLRP3 inflammasome [10]. However, it remains uncertain whether the PI3K/AKT pathway regulates the activation of the NLRP3 inflammasome and influences pyroptosis in HFD-induced OCM mice.

Currently, the prevention and treatment of OCM primarily focuses on lifestyle modifications, pharmacological interventions, and surgical procedures [11]. These approaches are effective in reducing body weight and alleviating cardiac burden but still exhibit limitations in preventing disease progression and promoting cardiac health, while exercise represents a low-risk, non-pharmacological intervention with distinctive advantages and potential for the treatment of OCM. It is reported that resistance exercise improves myocardial fibrosis in mice with myocardial infarction by inhibiting oxidative stress and cellular apoptosis levels [12]. Studies also found that aerobic exercise could ameliorate the progression of diabetic cardiomyopathy by modulating pyroptosis levels and mitigating adverse cardiac remodeling caused by inflammatory cell death [13]. Additionally, aerobic exercise alleviated cardiac toxicity by activating inflammasomes to inhibit pyroptosis, thereby improving cardiac dysfunction and hypotension in mice with doxorubicin-induced cardiomyopathy [14]. In summary, aerobic exercise is recognized as an effective intervention for alleviating obesity-induced cardiac injury. Nevertheless, the underlying mechanisms of aerobic exercise in the treatment of OCM remain unclear and require further investigation.

Therefore, middle-aged mouse models of OCM are generated through HFD feeding. The models are subjected to an 8-week intervention comprising aerobic exercise and the PI3K inhibitor LY294002. It is hypothesized that aerobic exercise may reduce the activation of NLRP3 inflammasomes by inhibiting the PI3K/AKT signaling pathway, thereby reducing inflammatory responses and improving cardiac structure and function, thereby alleviating OCM pyroptosis.

## 2. Results

### 2.1. High-Fat Diet Feed Established Middle-Aged OCM Mice

**Figure 1:**
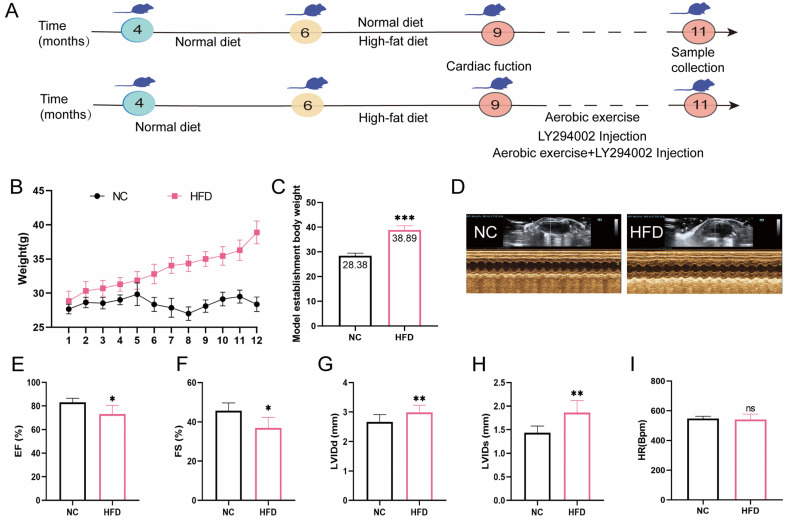
Effects of HFD on body weight and cardiac function in middle-aged mice. (**A**) Detailed experimental timeline including model establishment, exercise intervention, administration of LY294002, and sample collection. (**B**) Statistical analysis of body weight changes in mice over 12 weeks of feeding to establish an obesity model. (**C**) After 12 weeks of feeding on different diets, the body weight of mice was measured. (**D**) Representative echocardiographic images of the left ventricle. (**E**–**I**) Echocardiographic results and assessment of cardiac function. All data are expressed as mean ± standard deviation (M ± SD) (*n* = 8). * *p* < 0.05, ** *p* < 0.05, *** *p* < 0.001 compared with the NC group. ns: no significant difference between the HFD group compared to the NC group.

To evaluate the effects of a HFD on body weight, cardiac structure, and function in mice, we regularly monitored body weight. Cardiac ultrasound was conducted to assess changes in cardiac structure and function (Figure 1A,D). The results showed that, compared with the NC group, the body weight of mice in the HFD group showed a continuous upward trend (Figure 1B). After 12 weeks of feeding, statistical analysis showed that there was a significant difference in body weight between the two groups, and the body weight of the HFD group was significantly higher than that of the NC group by 20% (Figure 1C). Cardiac ultrasound analysis further revealed the effect of HFD on cardiac structure and function in mice (Figure 1E–H). Compared with the NC group, LVIDd and LVIDs in the HFD group significantly increased, and EF and FS significantly decreased, but there was no significant difference in heart rate. These results indicate that a 12-week high-fat diet successfully constructed a mouse model of OCM.

### 2.2. Aerobic Exercise Alleviated Weight and Blood Lipid Level in Middle-Aged OCM Mice

**Figure 2:**
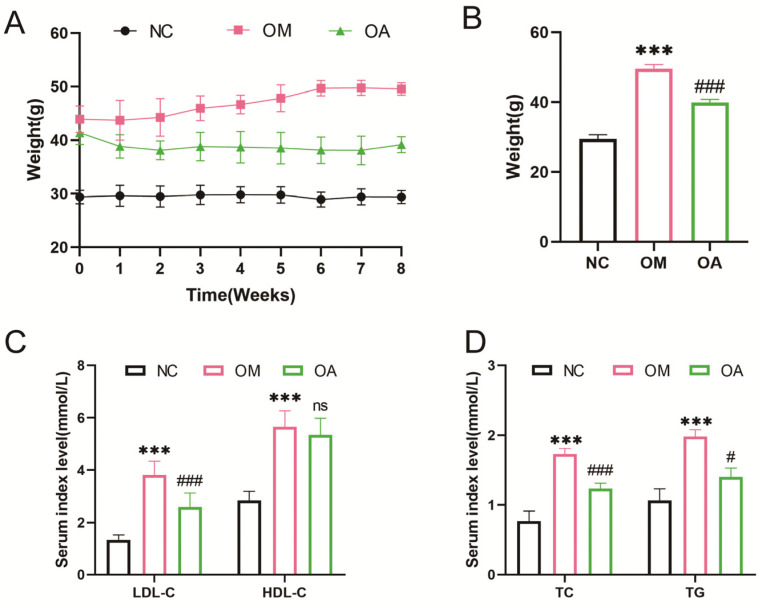
Effects of aerobic exercise on body weight and lipid levels in OCM mice. (**A**) Changes in body weight of mice during the intervention. (**B**) Body weight of mice in each group after intervention. (**C**) Serum levels of LDL-C and HDL-C in mice in each group after intervention. (**D**) Serum levels of TC and TG in mice in each group after intervention. All data are expressed as mean ± standard deviation (M ± SD) (*n* = 6). *** *p* < 0.001 versus to NC group; ^#^ *p* < 0.05, ^###^ *p* < 0.001 versus to OM group. ns: no significant.

The experiment revealed a significant reduction in body weight among mice in the OA group during the intervention period, with a significant reduction observed in the eighth week (Figure 2A,B). Additionally, when serum lipid levels were examined, it was found that the LDL-C, HDL-C, TC, and TG levels in the OM group were significantly higher than those in the NC group. After the 8-week aerobic exercise intervention, LDL-C, TC, and TG levels in the OA group were significantly lower than those in the OM group. However, the reduction in the HDL-C level was not significant (Figure 2C,D). These findings suggest that 8 weeks of aerobic exercise led to a decrease in body weight and an improvement in dyslipidemia.

### 2.3. Aerobic Exercise Ameliorated Myocardial Fibrosis and Cardiac Dysfunction in Middle-Aged OCM Mice

**Figure 3:**
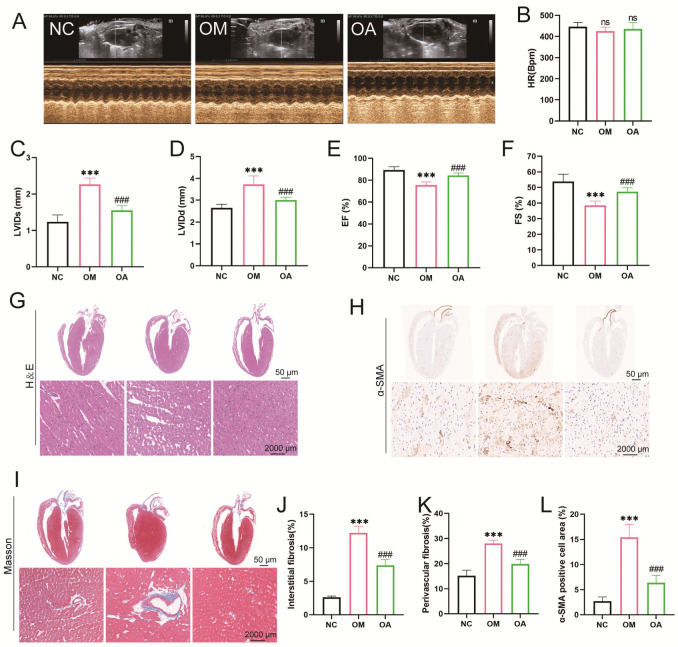
Effects of aerobic exercise on myocardial structure and function in mice. (**A**) Representative echocardiogram of the left ventricle. (**B**) Changes in HR. (**C**) Changes in LVIDd. (**D**) Changes in LVIDs. (**E**) Changes in EF. (**F**) Changes in FS. Representative images of H&E staining (**G**), α-SMA immunohistochemical staining (**H**), and Masson staining (**I**) from each group (scale bar 50 μm and 2000 μm). (**J**) Quantification of the percentage of areas staining positive for interstitial fibrosis. (**K**) Quantification of the percentage of areas staining positive for perivascular fibrosis. (**L**) Quantification of the percentage of α-SMA-positive cell area. All data are expressed as mean ± standard deviation (M ± SD) (*n* = 6). *** *p* < 0.001 versus to NC group; ^###^ *p* < 0.001 versus to OM group. ns: no significant.

Echocardiographic analyses showed that LVIDd and LVIDs were significantly greater, while EF and FS were significantly lower in mice’s OM group than in the NC group. After 8 weeks of aerobic exercise intervention, LVIDd and LVIDs were significantly reduced, and EF and FS significantly increased in the OA group compared with the OM group. However, no statistical difference was seen in HR (Figure 3A–F). Observation of myocardial histopathological changes by HE staining revealed that compared with the NC group, cardiomyocytes in the OM group presented typical pathological features such as widened cell gaps, disordered arrangement, and increased heterogeneity of cell nuclear morphology. Notably, the above myocardial structural damage was significantly improved in the OA group after 8 weeks of aerobic exercise intervention (Figure 3G). Quantitative analysis of Masson staining showed that the area of myocardial interstitial fibrosis and perivascular collagen deposition significantly increased in the OM group compared to the OC group. In contrast, myocardial fibrosis was significantly reduced in the OA group after aerobic exercise intervention (Figure 3I–K). In addition, immunohistochemical quantification showed that the α-SMA-positive area of myocardial tissue was reduced in the OA group compared with the OM group (Figure 3H,L). The above evidence suggests that 8 weeks of regular aerobic exercise effectively improves myocardial structural remodeling and restores cardiac pumping function.

### 2.4. Aerobic Exercise Suppressed PI3K/AKT Activation and Activation of Pyroptosis

**Figure 4:**
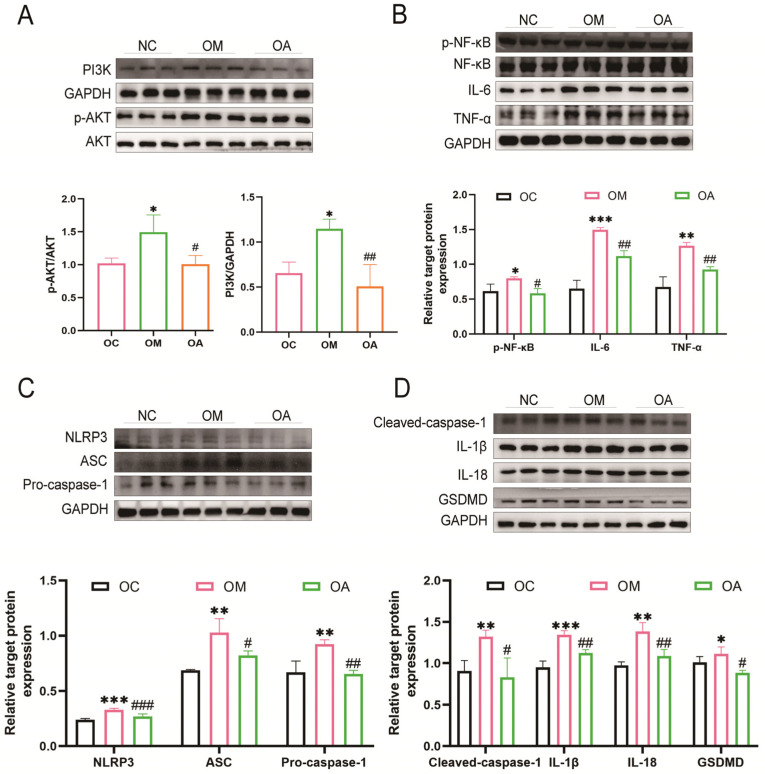
Effects of aerobic exercise on the PI3K/AKT and pyroptosis signaling pathway. (**A**) Western blot showing protein levels and quantitative analysis of PI3K, p-AKT, and AKT in different groups. (**B**) Western blot showing protein levels and quantitative analysis of p-NF-κB, p-NF-κB, IL-6, and TNF-α in different groups. (**C**) Western blot showing protein levels and quantitative analysis of NLRP3, ASC, and Pro-caspase-1 in different groups. (**D**) Western blot showing protein levels and quantitative analysis of Cleaved-caspase-1, IL-1β, IL-18, and GSDMD in different groups. All data are expressed as mean ± standard deviation (M ± SD) (*n* = 3). * *p* < 0.05, ** *p* < 0.01, *** *p* < 0.001 versus the NC group; ^#^ *p* < 0.05, ^##^ *p* < 0.01, ^###^ *p* < 0.001 versus the OM group.

To investigate the mechanism of aerobic exercise in alleviating OCM, we examined the PI3K/AKT pathway and its downstream inflammatory factors. Our findings revealed that the PI3K/AKT signaling pathway and relative factors p-NF-κB, NF-κB, IL-6, and TNF-α were activated in the OM group. Conversely, following the exercise intervention, the levels of these factors were down-regulated effectively in the OA group (Figure 4A,B). To further understand the mechanism of myocardial injury in an inflammatory state, we examined the NLRP3 inflammasome-mediated pyroptosis pathway. A significant elevation in the levels of NLRP3, ASC, Pro/Cleaved-caspase-1, IL-1β, IL-18, and GSDMD were shown in the OM group, which were markedly reduced following an 8-week exercise intervention (Figure 4C,D). In conclusion, our results demonstrated that aerobic exercise inhibited the PI3K/AKT signaling pathway and concomitantly suppressed the activation of pyroptosis.

### 2.5. Inhibition of PI3K/AKT Signaling Pathway Attenuates Lipid Deposition in Middle-Aged Obese Cardiomyopathy Mice

**Figure 5:**
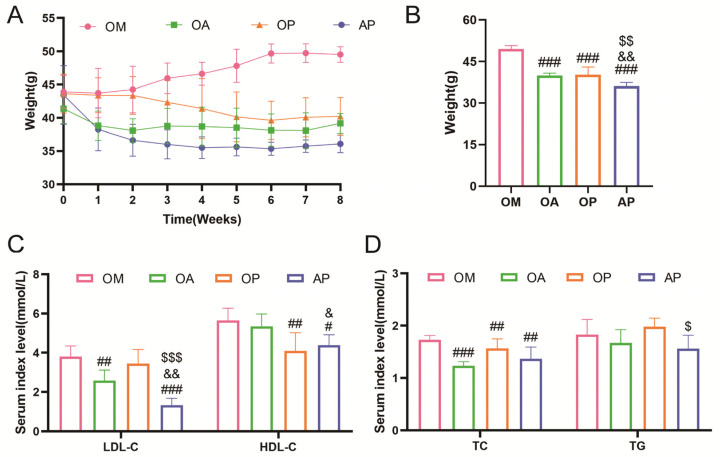
Effects of PI3K inhibitors on lipid metabolism in mice. (**A**) Body weight of mice in each group during the intervention. (**B**) Body weight of mice in each group after intervention. (**C**,**D**) Serum expression levels of LDL, HDL, TG, and TC in each group of mice. All data are expressed as mean ± standard deviation (M ± SD) (*n* = 6). ^#^ *p* < 0.05, ^##^ *p* < 0.01, ^###^ *p* < 0.001 versus the OM group; ^&^ *p* < 0.05, ^&&^ *p* < 0.01 versus the OA group; ^$^ *p* < 0.05, ^$$^ *p* < 0.01, ^$$$^ *p* < 0.001 versus the OP group.

To further investigate the role of PI3K/AKT signaling in OCM disease, we introduced groups treated with a PI3K inhibitor and a combination of exercise and PI3K inhibitor. During the intervention period, the OM group exhibited an increase in body weight, while the body weights of mice in the OA, OP, and AP groups were significantly lower than those in the OM group. Additionally, the AP group had significantly lower body weights compared to the OA and OP groups (Figure 5A,B). Blood lipid levels were markedly reduced in both the OA and AP groups, with the AP group showing a more pronounced effect than the OA and OP groups. Conversely, the OP and AP groups exhibited a notable decline in HDL-C levels, with the AP group exhibiting a more pronounced effect than the OA group. Furthermore, OA, OP, and AP groups showed a significant reduction in TC levels compared to the OM group. However, the decline in TG levels was not statistically significant across all groups, with only the AP group demonstrating a more pronounced reduction than the OP group (Figure 5C,D). These results suggest that inhibiting PI3K signaling could serve to alleviate the abnormal lipid metabolism observed in OCM mice.

### 2.6. Inhibition of PI3K Signaling Alleviates Impaired Myocardial Structure and Function in Middle-Aged OCM Mice

**Figure 6:**
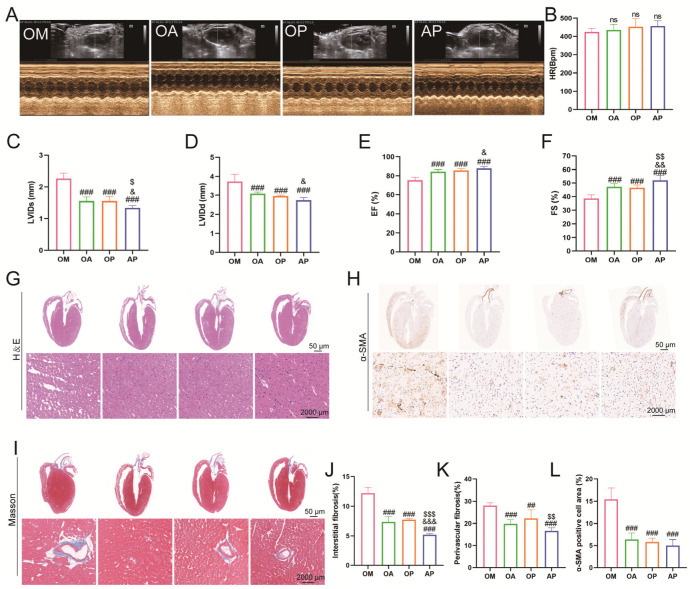
Effects of inhibition of PI3K signaling on cardiac structure and function in mice. (**A**) Representative echocardiogram of the left ventricle. (**B**) Changes in HR. (**C**) Changes in LVIDd. (**D**) Changes in LVIDs. (**E**) Changes in EF. (**F**) Changes in FS. Representative images of H&E staining (**G**), α-SMA immunohistochemical staining (**H**), and Masson staining (**I**) from each group (scale bar 50 μm and 2000 μm). (**J**) Quantification of the percentage of areas staining positive for interstitial fibrosis. (**K**) Quantification of the percentage of areas staining positive for perivascular fibrosis. (**L**) Quantification of the percentage of α-SMA-positive cell area. All data are expressed as mean ± standard deviation (M ± SD, *n* = 6). ^##^ *p* < 0.01, ^###^ *p* < 0.001 versus the OM group; ^&^ *p* < 0.05, ^&&^ *p* < 0.01, ^&&&^ *p* < 0.01 versus the OA group; ^$^ *p* < 0.05, ^$$^ *p* < 0.01, ^$$$^ *p* < 0.001 versus the OP group. ns: no significant.

To further investigate the regulatory mechanism of the PI3K signaling pathway, we systematically evaluated the intervention effect of LY294002 on cardiac function. Quantitative echocardiographic analysis showed that, compared with the OM group, the LVIDd, LVIDs, EF, and FS of mice in the OA, OP, and AP groups were significantly improved, with the most obvious effect in the AP group. At the same time, there was no statistically significant difference in HR between the groups (Figure 6A–F). HE staining pathological analysis showed that the OM group presented typical pathological myocardial remodeling features, including widening of the gap of the cardiac myocytes, disordered arrangement, and increased heterogeneity of cell nuclear morphology (Figure 6G). Disorders and increased heterogeneity of cell nuclear morphology (Figure 6G), and the pathological state of myocardium, improved in all groups of mice after intervention. Quantification by Masson staining revealed that the percentage of myocardial interstitial fibrosis area was significantly reduced in all groups of mice after intervention, and the interstitial fibrosis was most significantly improved in the AP group (Figure 6I,J). At the same time, the perivascular collagen volume percentage was also significantly reduced in all groups of mice, and the decrease was also most significant in the AP group (Figure 6K). Using immunohistochemistry, quantitative analysis of α-SMA-positive cell area revealed that the percentage of area staining positive in the heart was significantly decreased in all groups of mice compared with mice in the OM group (Figure 6H,L). These findings indicate that the inhibition of PI3K signaling could mitigate myocardial structural and functional disturbances.

### 2.7. Exercise Synergises with PI3K Inhibitors to Inhibit Pyroptosis in Middle-Aged OCM Mice

**Figure 7:**
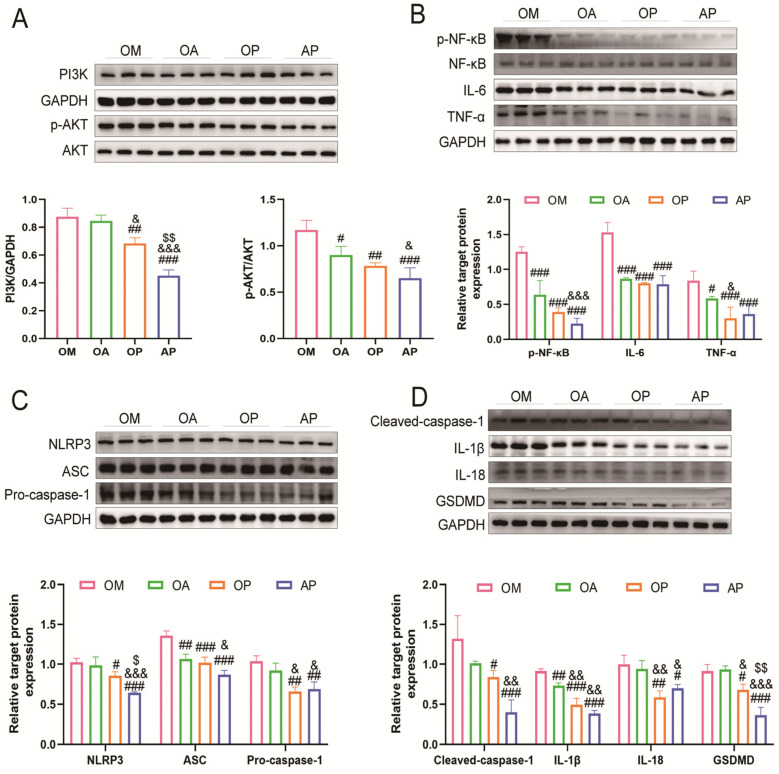
The role of exercise and PI3K inhibitors on pyroptosis. (**A**) Western blot showing protein levels and quantitative analysis of PI3K, p-AKT, and AKT in different groups. (**B**) Western blot showing protein levels and quantitative analysis of p-NF-κB, p-NF-κB, IL-6, and TNF-α in different groups. (**C**) Western blot showing protein levels and quantitative analysis of NLRP3, ASC, Pro-caspase-1 in different groups. (**D**) Western blot showing protein levels and quantitative analysis of Cleaved-caspase-1, IL-1β, IL-18, and GSDMD in different groups. All data are expressed as mean ± standard deviation (M ± SD, *n* = 3). ^#^ *p* < 0.05, ^##^ *p* < 0.01, ^###^ *p* < 0.001 versus the OM group; ^&^ *p* < 0.05, ^&&^ *p* < 0.01, ^&&&^ *p* < 0.01 versus the OA group; ^$^ *p* < 0.05, ^$$^ *p* < 0.01 versus the OP group.

To further explore the mechanism by which aerobic exercise in alleviates OCM, we included a PI3K inhibitor group and an exercise combined with inhibitor group in the intervention. After 8 weeks, the expression levels of PI3K, AKT, p-NF-κB, NF-κB, IL-6, and TNF-α were significantly reduced in the OA, OP, and AP groups compared to the OM group. Notably, the AP group exhibited the most pronounced decrease (Figure 7A,B). Additionally, analysis of the NLRP3 inflammasome-mediated pyroptosis pathway revealed a significant reduction in ASC and IL-1β levels in the OA group compared to the OM group. The OP and AP groups also demonstrated significant declines in NLRP3, ASC, Pro/Cleaved-caspase-1, IL-1β, IL-18, and GSDMD levels, with the AP group showing the most pronounced effects (Figure 7C,D). In summary, exercise intervention and PI3K/AKT signaling inhibition have synergistic effects through a pyroptosis mechanism, and both their independent and combined effects significantly ameliorate OCM.

## 3. Discussion

OCM is a serious condition characterized by cardiac functional, morphological, and metabolic abnormalities caused by obesity [15]. It worsens the burden of heart failure and atrial fibrillation, typically presenting as dilated cardiomyopathy [16]. Currently, the standard treatment involves the administration of lipid-lowering drugs, but no specific therapy exists for OCM [17]. Aerobic exercise has proven effective in reducing body weight, improving cardiac function, and alleviating obesity-related conditions [18]. Prior research showed that an 8-week aerobic exercise program significantly improved EF and FS levels, alleviating cardiac dysfunction, fibrosis, and lipid deposition [19]. Another study has demonstrated that swimming exercise significantly reduces myocardial fibrosis, cardiac dysfunction, and lipid accumulation in HFD-induced lipotoxic cardiomyopathy [20]. Similarly, in terms of regulating lipid levels, our research indicates that 8 weeks of aerobic exercise decreases body weight and alleviates abnormal blood lipid levels in obese mice. It also improves LVIDd and LVIDs, reversing EF and FS to alleviate cardiac dysfunction. Additionally, myocardial fibrosis is a hallmark of OCM [16], and 8 weeks of aerobic exercise could also ameliorate myocardial cell disarray and reduce collagen deposition and fibroblast remodeling, thereby mitigating the occurrence of myocardial fibrosis. While exercise can alter epigenetic changes induced by OCM, the molecular mechanisms behind its protective effects on obesity-induced cardiomyopathy remain unclear.

**Figure 8:**
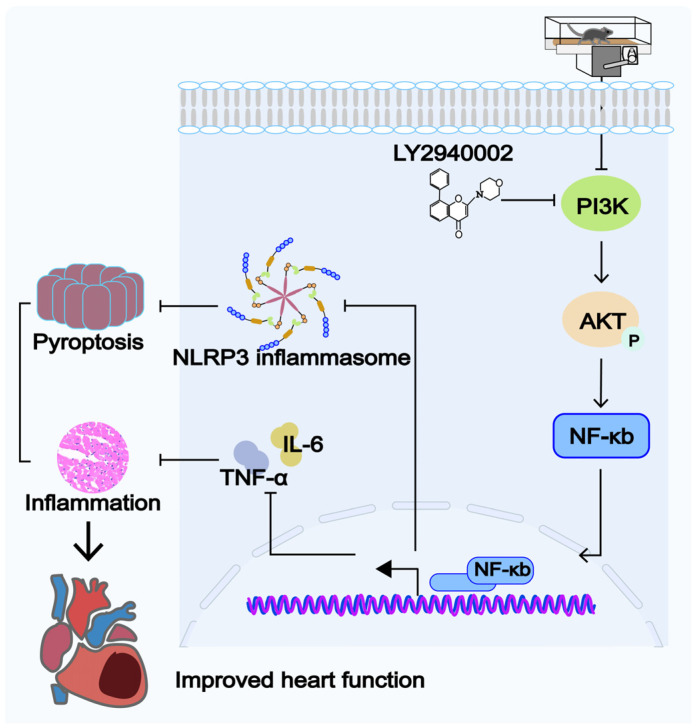
Aerobic exercise inhibits the activation of NLRP3 inflammasome by suppressing the PI3K/AKT signaling pathway, thereby alleviating the pyroptosis and inflammatory response of cardiomyocytes in mice with OCM.

This study demonstrated that long-term HFD feeding induced OCM in middle-aged mice, leading to myocardial structural and functional impairments. Aerobic exercise intervention could mitigate HFD-induced OCM and alleviate the damage to myocardial structure and function. Mechanistically, aerobic exercise participated in the pathological process of the disease by inhibiting the PI3K/AKT pathway and NLRP3-induced pyroptosis. Further research revealed that the inhibition of PI3K (LY294002) reduced inflammatory responses and suppressed the activation of pyroptosis significantly (Figure 8). These findings provide new insights into the understanding and treatment of OCM.

The pathogenesis of obesity-induced cardiomyopathy resulting from a HFD includes autophagy, mitochondrial dysfunction, endoplasmic reticulum stress, oxidative stress, and inflammation [16,21]. In obesity, white adipose tissue produces increased levels of pro-inflammatory adipokines, exacerbating cardiovascular dysfunction [22]. In addition, studies have found that fatty acids could activate the NF-κB signaling pathway, leading to increased expression of pro-inflammatory cytokines such as IL-6 and TNF-α [23]. The canonical NF-κB pathway is initiated by pattern recognition receptors (PRRs), with NLRP3 inflammasomes acting as innate immune sensors in the NOD-like receptor (NLR) family. NF-κB activation triggers a cascade, recruiting and activating caspase-1, which catalyzes the conversion of IL-1β and IL-18 into their mature forms and cleaves GSDMD to generate N-terminal fragments that form transmembrane pores, promoting inflammation and pyroptosis [21]. A rise in body weight in mice is accompanied by NLRP3-inflammasome activation in the heart and other organs [24]. Recent research emphasizes the significant roles of NLRP3 inflammasome and pyroptosis in obesity-related cardiomyopathy [25]. Anti-obesity measures such as caloric restriction and exercise were shown to lower levels of adipose NLRP3 and suppress inflammation [26]. Notably, treadmill exercise has been shown to alleviate cardiac pyroptosis and inflammation in HFD mice [19]. Similarly, our research confirms that 8 weeks of treadmill aerobic exercise could ameliorate cardiac inflammation and pyroptosis in mice with OCM. In conclusion, these findings suggest that aerobic exercise benefits OCM by inhibiting pyroptosis and inflammation.

The PI3K/AKT signaling pathway is a key regulator of cellular growth, proliferation, and metabolism [27]. Accumulating evidence highlights its critical role in cardiovascular diseases. Wang’s research revealed that traditional Chinese medicine could ameliorate cardiac remodeling and inflammation in rats with myocardial infarction by inhibiting the PI3K/AKT signaling pathway [28]. Moreover, PI3K/AKT inhibition has been shown to regulate myocardial hypertrophy and fibrosis, thereby alleviating pressure overload-induced cardiac remodeling [29]. In diabetic rats, high-intensity interval training alleviates cardiac hypertrophy and apoptosis through downregulation of the PI3K/AKT pathway. Our study demonstrated that 8 weeks of aerobic exercise mitigated HFD-induced OCM by inhibiting PI3K/AKT signaling. Interestingly, a study found that 8 weeks of aerobic exercise attenuated HFD-induced myocardial inflammation and fibrosis by activating the PI3K/AKT pathway [30], whereas 4 weeks of aerobic exercise activated the PI3K/AKT signaling pathway to alleviate insulin resistance in diabetic rats [31]. This discrepancy may be related to the intrinsic differences between the mice. Unlike previous studies of using 8-week-old mice to establish OCM models [32], we employed 24-week-old mice to establish a model of middle-aged obese OCM. After long-term aerobic exercise, the mice exhibited improved cardiac function. Age influences intrinsic states, as young mice are more responsive to physiological changes and more resilient to cardiac dysfunction [33]. Age-related changes in cardiac contractile and regulatory proteins have differential effects on cardiac insufficiency [34]. Additionally, the impaired cardiac phenotypes in middle-aged mice more closely recapitulate the pathological hallmarks of human obesity-related cardiomyopathy compared to younger counterparts [35]. The research found that aerobic exercise attenuates HFD-induced cardiac pyroptosis and inflammation by stimulating interferon genes’ (STING)-NLRP3 signaling axis [19]. Exercise and enhanced PI3K could delay or prevent progress of cardiomyopathy [36]. However, the study of the PI3K/AKT signaling pathway through NLRP3 inflammasome in OCM has not been fully elucidated. To further clarify the role of PI3K/AKT signaling in OCM, we utilized LY294002 and found that inhibition of PI3K/AKT signaling effectively alleviated myocardial lipid accumulation, fibrosis, and dysfunction. In summary, these findings indicate that aerobic exercise inhibited pyroptosis in OCM by regulating NLRP3 inflammasome crosstalk through the PI3K/AKT.

Recent research has demonstrated that the PI3K/AKT signaling pathway could activate NF-κB and promote its nuclear translocation through phosphorylation. This pathway is closely linked to inflammation and acts as an upstream target for NLRP3 inflammasome factor activation [37,38]. In studies of DCM [39], autoimmune prostatitis [40], cerebral ischemia [41], and other diseases, modulation of the PI3K/AKT pathway has been shown to inhibit NLRP3 activation and regulate downstream inflammatory factor expression. NLRP3 inflammasomes are also crucial regulators of pyroptosis. In aging-related brain injury [10] and cerebral hemorrhage [42], inhibiting NLRP3-mediated pyroptosis could be achieved through modulation of the PI3K/AKT pathway. Studies have confirmed that in DCM, LY294002 injection could attenuate myocardial fibrosis and improve cardiac function in rats [43]. However, the role of the PI3K/AKT pathway in regulating NLRP3-induced pyroptosis in OCM remains unclear. Therefore, we injected PI3K inhibitor LY294002 into the OCM mice and observed that, compared to the OM group, NLRP3-induced pyroptosis signaling was inhibited in the OCM model with OP group, with a significant reduction in the expression of inflammatory factors. These findings suggest that the PI3K/AKT pathway regulates pyroptosis in OCM. Additionally, studies have indicated that aerobic exercise could alleviate chondrocyte inflammation by inhibiting the PI3K/AKT/NF-κb signaling pathway, thereby improving osteoarthritis-related pyroptosis [44]. To further investigate the synergetic effect, we combined aerobic exercise and LY294002. The results revealed that the AP group exhibited more significant improvements compared to the OA and OP groups in lipid levels, myocardial fibrosis, and cardiac dysfunction. Furthermore, the PI3K/AKT pathway was significantly suppressed in the AP group, accompanied by marked inhibition of pyroptosis and inflammatory signals. Increased activation of class IA PI3Ks and their downstream effector AKT in cancer and the immune system has led to significant development of inhibitors targeting the PI3K pathway [45]. Research has demonstrated that a PI3K knockout mouse model provides intuitive evidence that a specific small molecule compound can activate PI3K, protecting the heart and promoting neuronal regeneration [46]. Furthermore, myeloid-specific targeted knockout of PI3K, compared to the broad use of inhibitors, significantly reduces off-target effects in non-malignant tissues and offers a clearer elucidation of the PI3K/AKT signaling pathway mechanisms [47]. Notably, research has provided an example where small-molecule inhibitors and genetic knockout do not produce the same phenotype in vivo, and small-molecule inhibitors had unique advantages in achieving substrate-selective inhibition [48]. Therefore, in-depth exploration of the role of PI3K gene knockout in improving OCM will be key to future research.

### Limitations

Our study still has some limitations. The findings of this study were based on the addition of the PI3K inhibitor LY294002. If specific knockout mice are used, the expected effect will be more significant. Further research is necessary to examine this topic and enhance the findings of this study. In addition, it is important to include more diverse exercise intervention programs to clarify the role of exercise in OCM.

## 4. Materials and Methods

### 4.1. Animal Groupings and Intervention Programs

All experimental protocols were reviewed and approved by the Experimental Management Centre of Wuhan Sports University (S0087-20221123-01). Animal husbandry and interventions were carried out in a specific pathogen-free environment in the laboratory animal laboratory of Wuhan Sports University with a 12 h light/dark cycle and controlled temperature and humidity. A number of 16-week-old male C57BL/6J mice (purchased from Henan Skebest Biotechnology Co., Ltd., Zhengzhou, China. 25 ± 1.05 g, Certificate No. 410983231100044161) were initially fed a standard diet for 8 weeks. They were then randomly divided into two groups: the control group (NC, *n* = 15), which continued on the standard diet, and the high-fat diet group (HFD, *n* = 65). The standard diet contained 4% fat, while the high-fat diet contained 60% fat.

After 12 weeks of HFD feeding, the HFD group were screened according to established criteria to identify those that met the criteria for the OCM model. The weight of the model group was significantly higher than that of the normal control group, and the weight gain was more than 20 % of the average value of the normal group. Cardiac structure and function were also detected (animals in the model group should have ventricular hypertrophy, cardiac diastolic or systolic dysfunction) [49]. These mice were then randomly assigned to the model group (OM, *n* = 12), the aerobic exercise group (OA, *n* = 12), the PI3K inhibitor LY294002 group (OP, *n* = 12), or the aerobic exercise combined with PI3K inhibitor LY294002 group (AP, *n* = 12). Mice in each group were sampled after 8 weeks of intervention.

At the onset of the training protocol, the OA and AP groups underwent a 5-day treadmill adaptation program. On Day 1, the mice ran for 30 min at a speed of 12 m/min. The intensity was progressively increased by 1 m/min each day, and the duration was extended by 10 min daily until the adaptation phase was completed. Following this period, the mice rested for 2 days before beginning an 8-week formal aerobic exercise regimen. This regimen consisted of 5 weekly sessions, each lasting 60 min, with a running speed maintained at 16 m/min. Mice in the OP and AP groups received LY294002 (S1737-25 mg, from Shanghai, China) at a dosage of 0.5 mg/kg, administered intraperitoneally, five days a week for 8 weeks, in accordance with the manufacturer’s instructions.

### 4.2. Lipid Level Detection

Blood samples were collected and processed to obtain the upper serum layer. Total cholesterol (TC, A111-1-1, Nanjing, China), triglycerides (TG, A110-1-1, Nanjing, China), high-density lipoprotein cholesterol (HDL, A112-1-1, Nanjing, China), and low-density lipoprotein cholesterol (LDL, A113-1-1, Nanjing, China) were detected to calculate the levels of each index in mouse serum.

### 4.3. Echocardiography

The mice were anesthetized by inhalation of isoflurane (R510-22-10, Shenzhen, China) at a concentration of 4% to induce anesthesia and 1.5% to maintain anesthesia. After supine fixation on the operating table and hair removal from the chest, echocardiography was performed using the M9Vet Ultrasound System (Shenzhen, China) in M-mode with parasternal long-axis views to determine ejection fraction (EF%), fractional shortening (FS%), left ventricular internal dimension at diastole (LVIDd), left ventricular internal dimension at systole (LVIDs), and heart rate (HR).

### 4.4. Histopathology

After perfusion of mouse hearts, whole hearts were fixed in paraformaldehyde for 48 h, then the hearts were removed, dehydrated, and embedded in paraffin, and 4-μm sections were cut with a slicer for hematoxylin–eosin (HE) and Masson staining, and observed and photographed under a microscope. To assess the degree of myocardial structural damage, collagen volume fraction was calculated using ImageJ (V1.8.0.112) software.

### 4.5. Immunohistochemistry

The left ventricular tissue was rinsed with cooled saline embedded in paraffin, sections were then dehydrated (4 μm), endogenous enzyme activity was inactivated, and they were then used for HE and immunohistochemistry staining. Following antigen repair using ethylenediaminetetraacetic acid antigen repair buffer (pH 9.0) and endogenous peroxidase with a 3% hydrogen peroxide solution, and blocking of non-specific binding sites using a 3% goat serum, the sections were incubated with primary rabbit antibodies against anti α-smooth muscle actin (α-SMA, 1:100) and incubated overnight at 4 °C. Sections were washed three times with PBS and incubated with anti-rabbit IgG secondary antibody. a. After being re-stained with haematoxylin for 3 min and washed with pure water, the sections were observed under a microscope and photographed for storage, and the average optical density values of the positive areas were analyzed using ImageJ software to assess the degree of fibrosis in the myocardial tissue.

### 4.6. Western Blot

An appropriate amount of left ventricular myocardial tissue was weighed, lysed, and sonicated to prepare protein samples. Protein samples were separated by sodium dodecyl sulfate–polyacrylamide gel electrophoresis (SDS-PAGE) and transferred to nitrocellulose membranes. The membranes were incubated with primary antibodies overnight at 4 °C in Tris-buffered saline with Tween-20 (TBST), after sealing with skimmed milk powder for 1 h. Primary antibodies included PI3K (#4249 1:1000 CST USA) (Danvers, MA, USA); p-Akt (#4060 1:2000 CST); AKT (#4685 1:1000 CST); NLRP3 (S4S87 1:1000 CST); ASC (#DF6304 1:500 AffinitY); pro-caspase-1 (#AF5418 1:1000 AffinitY); cleaved-caspase-1 (AF4005 1:2000 AffinitY); IL-1β (AF7017 1:500 AffinitY); IL-18 (DF6252 1:500 AffinitY); GSDMD (AF4012 1:2000 AffinitY); IL-6 (#02701 1:500 Proteintech) (Wuhan, China); TNF-α (AF7014 1:500 AffinitY); p-NF-κb (AF2006 1:500 AffinitY); and NF-κb (AF5006 1:500 AffinitY) (Melbourne, Australia). After TBST washing, the membrane was incubated with rabbit horseradish peroxidase-conjugated secondary antibody (1:2000, HUABIO, Wuhan, China) for 1.5 h at room temperature. The strips were washed with TBST, and proteins were detected using an enhanced chemiluminescence kit. Images were captured using a gel imaging system (Nikon DS-U3, Nikon, Tokyo, Japan) and quantitatively analyzed using ImageJ software.

### 4.7. Data Analysis

All data were expressed as mean ± standard deviation (M ± SD) and corresponding statistical analysis was conducted using GraphPad Prism software (Version 8.0) with paired-sample *t*-tests for normal distribution or Wilcoxon non-parametric paired-sample tests to complete analyses and generate images. Data were analyzed using Dunnett’s test to determine the significance of variance and then by one-way ANOVA. *p* < 0.05 was considered statistically significant.

## 5. Conclusions

In conclusion, this study confirms that aerobic exercise and LY294002 inhibit pyroptosis by inhibiting the PI3K/AKT signaling pathway from regulating NLRP3 inflammasome crosstalk, thereby improving OCM. Consequently, aerobic exercise is poised to emerge as a promising therapeutic strategy, holding significant importance for the prevention and treatment of obesity-related cardiomyopathy.

## Data Availability

The original contributions presented in this study are included in the article. Further inquiries can be directed to the corresponding author.
